# Supplementary material for: On age-specific selection and extensive lifespan beyond menopause
Source: R Soc Open Sci. 2020 May 6;7(5):191972. doi: 10.1098/rsos.191972 (PMC7277242; doi:10.1098/rsos.191972)
Supplement: Supplementary Figures [file rsos191972supp1.docx]

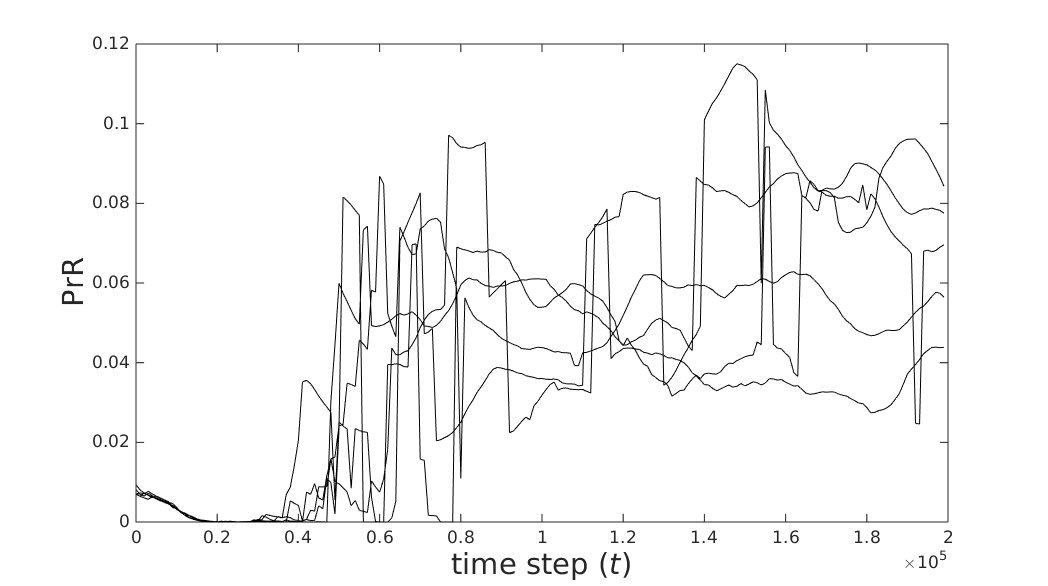


**Figure S1**. Post-reproductive representation (PrR) of the female individuals in the simulated populations of condition NULL. Each curve represents a distinct simulation.
